# Supplementary material for: Start codon variant in LAG3 is associated with decreased LAG-3 expression and increased risk of autoimmune thyroid disease
Source: Nat Commun. 2024 Jul 9;15:5748. doi: 10.1038/s41467-024-50007-7 (PMC11233504; doi:10.1038/s41467-024-50007-7)
Supplement: Supplementary file 1 — Supplementary Information [file 41467_2024_50007_MOESM1_ESM.pdf]

Supplementary Information

for the paper entitled „*Start codon variant in LAG3 is associated with decreased LAG-3 expression and increased risk of autoimmune thyroid disease*" by Saevarsdottir S et al.

Supplementary Figures (pages 2-7)

Supplementary Figure 1. Manhattan plot for the genome-wide association meta-analysis of autoimmune thyroid disease in study populations from Iceland, Finland, UK and USA (110,945 cases and 1,084,290 controls).

Supplementary Figure 2. Epstein-Barr virus immortalized B-cells from rs781745126-T carriers (CT) express less LAG-3 on surface and in cell supernatant than those from non-carriers (CC).

Supplementary Figure 3. Exhausted T-cells from rs781745126-T carriers express less LAG-3 and more PD-1.

Supplementary Figure 4. *LAG3* 5' UTR variant rs781745126-T does not associate with LAG3 mRNA expression in whole blood (n = 17,848; *P*=0.39, effect=-0.12SD).

Supplementary Figure 5. Predicted effect of coding variants that associate with autoimmune thyroid disease, in the *LAG3*, *ZAP70*, *ZNF800* and *ZNF429* genes, on structure of the encoded proteins.

Supplementary Figure 6. Flow cytometry gating strategy.

Supplementary Information (pages 8-11)

Methods for single-cell RNA sequencing and analyses.

Supplementary Figures

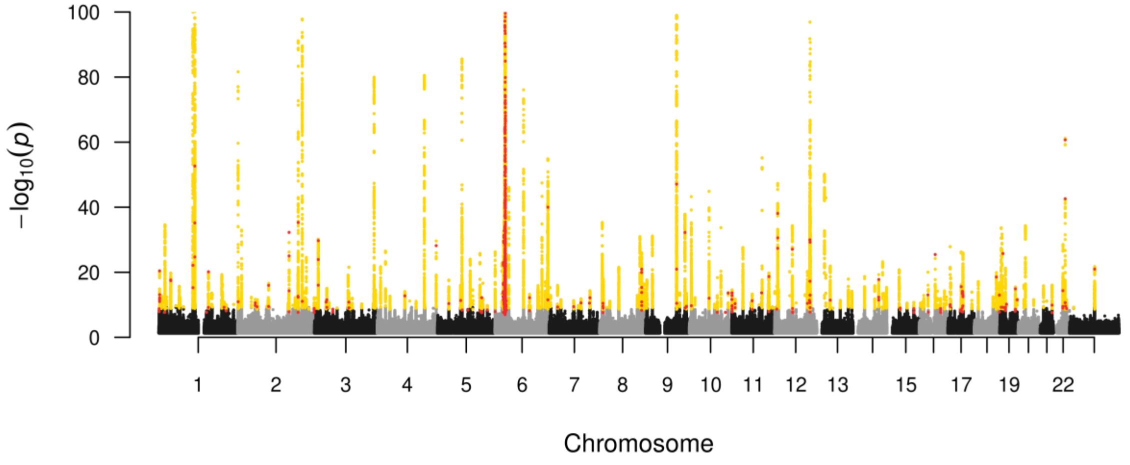

Supplementary Fig. 1. Manhattan plot for the genome-wide association meta-analysis of autoimmune thyroid disease in study populations from Iceland, Finland, UK and USA (110,945 cases and 1,084,290 controls). The GWAS was performed using logistic regression analysis assuming a multiplicative model, adjusting for year of birth, sex and origin (Iceland) or the first 20 (UK) or 4 principal components (USA). Sequence variants (N~56M variants) were split into five classes based on their genome annotation, and significance threshold for each class was adjusted for the number of variants in that class (e.g. lower thresholds for loss of function (high impact, marked in red) and missense variants (moderate impact, marked in orange), see also Methods and Supplementary Data 1.

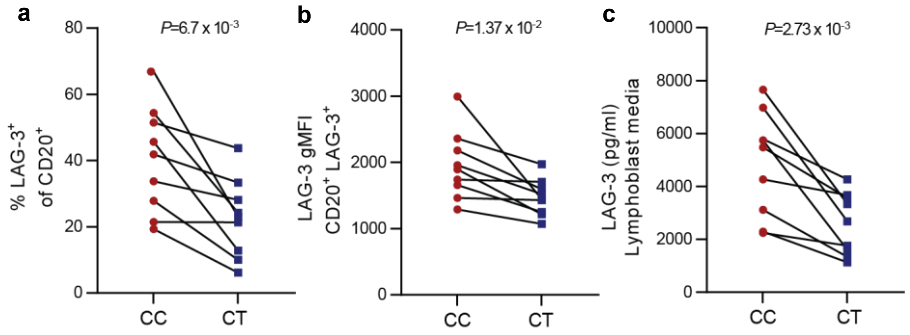

Supplementary Fig. 2. Epstein-Barr virus immortalized B-cells from rs781745126-T carriers (CT) express less LAG-3 on surface and in cell supernatant than those from non-carriers (CC). Heterozygous rs781745126-T carriers (n=9) were matched for age and sex to non-carriers (CC, n=9). Epstein-Barr virus immortalized B-cell lymphoblasts were incubated for 72 hours, supernatant collected and soluble LAG-3 measured by MSD (R-PLEX # F213Y-3, Meso Scale Diagnostics, see Methods). **(a)** LAG-3 frequency (%) and **(b)** surface expression intensity (geometric mean fluorescence intensity, gMFI). **(c)** LAG-3 levels in cell supernatant (lymphoblast media). Paired t-test with two-sided *P*-values was used to compare carriers (blue) and non-carriers (red) in (a)-(c).

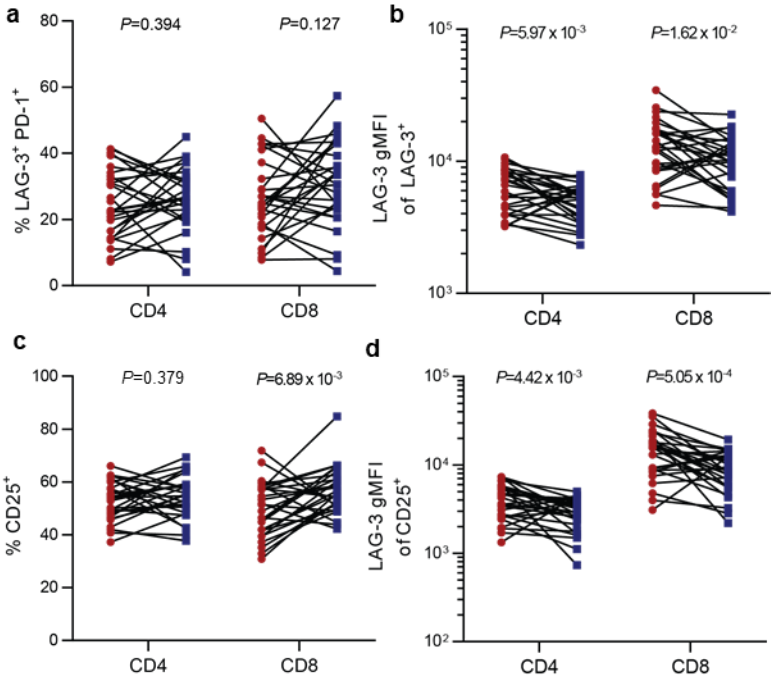

Supplementary Fig. 3. Exhausted T-cells from rs781745126-T carriers express less LAG-3 and more PD-1. PBMCs from heterozygous rs781745126-T carriers (CT, n=28) and matched non-carrier controls (CC, n=28) were stimulated for 48 hours with Staphylococcal enterotoxin B (SEB) to upregulate LAG-3 and PD-1 expression and induce exhaustion. **(a)** Frequency of LAG-3^+PD-1^+ double-positive CD4 and CD8 positive T-cells. **(b)** Surface expression intensity (geometric mean fluorescence intensity, gMFI) of LAG-3 on $CD4^+$ and $CD8^+$ T-cells that are $LAG3^+$. **(c)** Frequency of activated ($CD25^+$) $CD4^+$ and $CD8^+$ T-cells. **(d)** LAG-3 surface expression intensity of activated ($CD25^+$) $CD4^+$ and $CD8^+$ T-cells. Paired t-test with two-sided *P*-values was used to compare carriers (blue) and non-carriers (red) in (a)-(d).

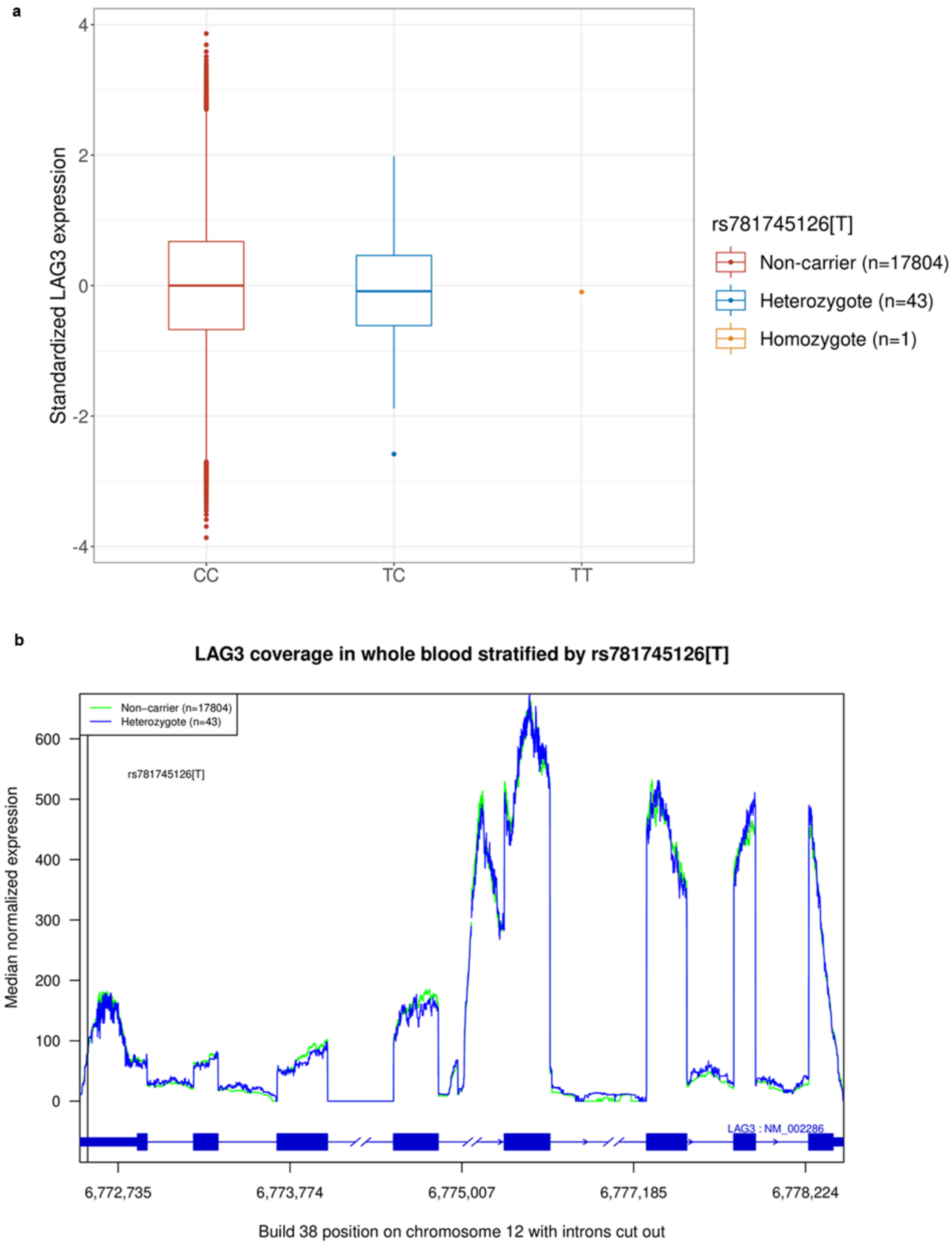

Supplementary Fig. 4. *LAG3* 5' UTR variant rs781745126-T does not associate with LAG3 mRNA expression in whole blood (n = 17,848; *P*=0.39, effect=-0.12SD. **(a)** Standardized LAG3 expression values stratified by the 5' UTR rs781745126-T variant. the distribution is shown by box-plots (outliers, 10th-90th percentile, interquartile range, median levels). **(b)** Median normalized LAG3 expression coverage stratified by the 5' UTR rs781745126-T variant, with the lone homozygote left out.

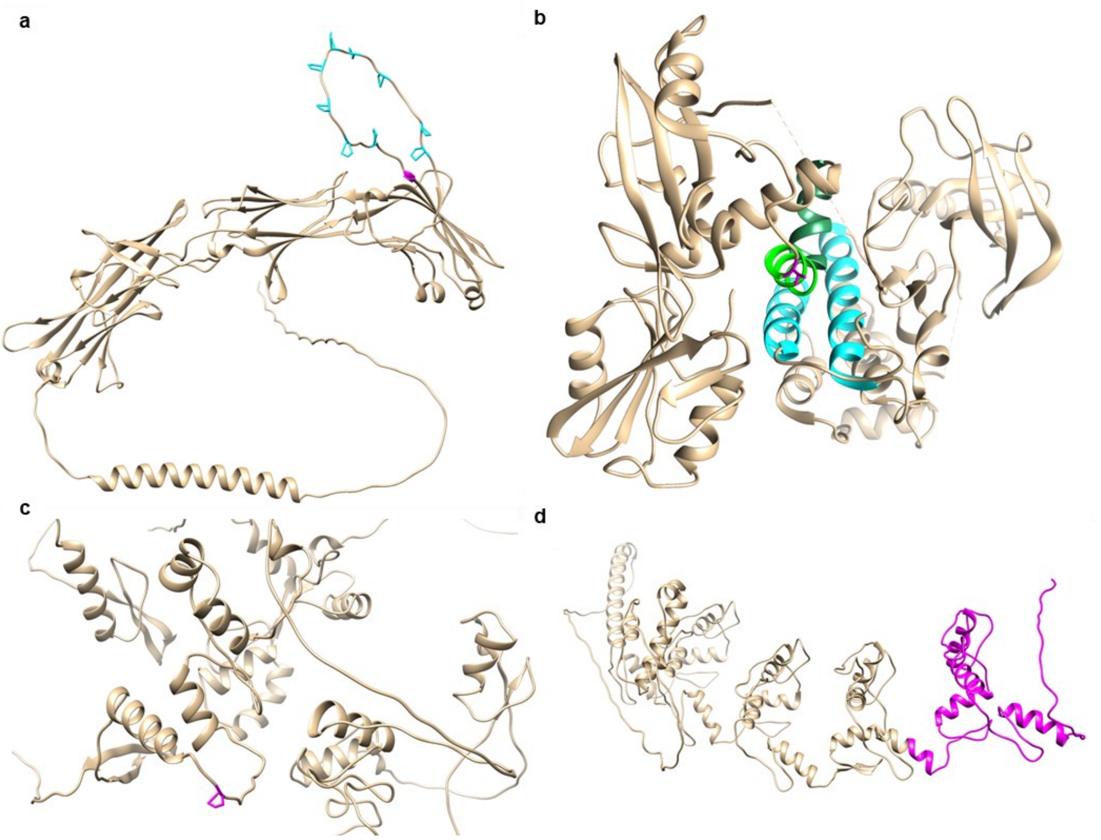

Supplementary Fig. 5. Predicted effect of coding variants that associate with autoimmune thyroid disease, in the *LAG3*, *ZAP70*, *ZNF800* and *ZNF429* genes, on structure of the encoded proteins. The structural models of the proteins are created with the Alphafold software for the reference (wild-type) proteins of LAG-3 (AF-P18627-F1), ZNF800 (AF-Q2TB10-F1) and ZNF429 (AF-Q86V71-F1) and the X-ray structure of ZAP-70 with PDB: 2ozo. **(a)** *LAG3* missense variant rs149722682 (p.Pro67Thr, magenta colour) is found in the D1 domain at the base of a large extra-loop structure (illustrating the prolines and the loop in cyan colour) that has been shown to be important for MHC-II binding and likely to affect the binding in a negative way. This is a proline rich loop which is not found in CD4 proteins and the prolines in the Pro67Thr domain are highly conserved among mammalians. **(b)** *ZAP70* missense variant rs145955907-T (Thr155Met, magenta colour) is in a highly conserved region of the ZAP-70 protein. It is predicted to be deleterious and replaces the hydrophilic threonine amino acid with hydrophobic methionine in an alpha helix referred to as L3 (green colour) in an inter-SH2 linker, which interacts with alpha helix I and E (cyan) from the kinase domain (ref: Deindl et al. 2007), which contains tyrosine residues that are phosphorylated during activation. Thr155Met is thereby likely to affect the protein structure and the kinase function of ZAP-70. **(c)** *ZNF800* missense variant rs62621812-A (p.Pro103Ser, magenta) is found in a disordered loop in between two alpha helices. The model confidence in this area is very low, but it is in a conserved region, and the proline amino-acid is in general conserved among mammalians due to its unique structure and mutations involving proline do often affect the protein structure. **(d)** *ZNF429* frameshift variant rs199679715-C occurs after 526 amino acids and results in a replacement of 148 amino acids with 166 other amino acids including 5.5 of 18 zinc-finger motifs (22% of the original protein, coloured in magenta).

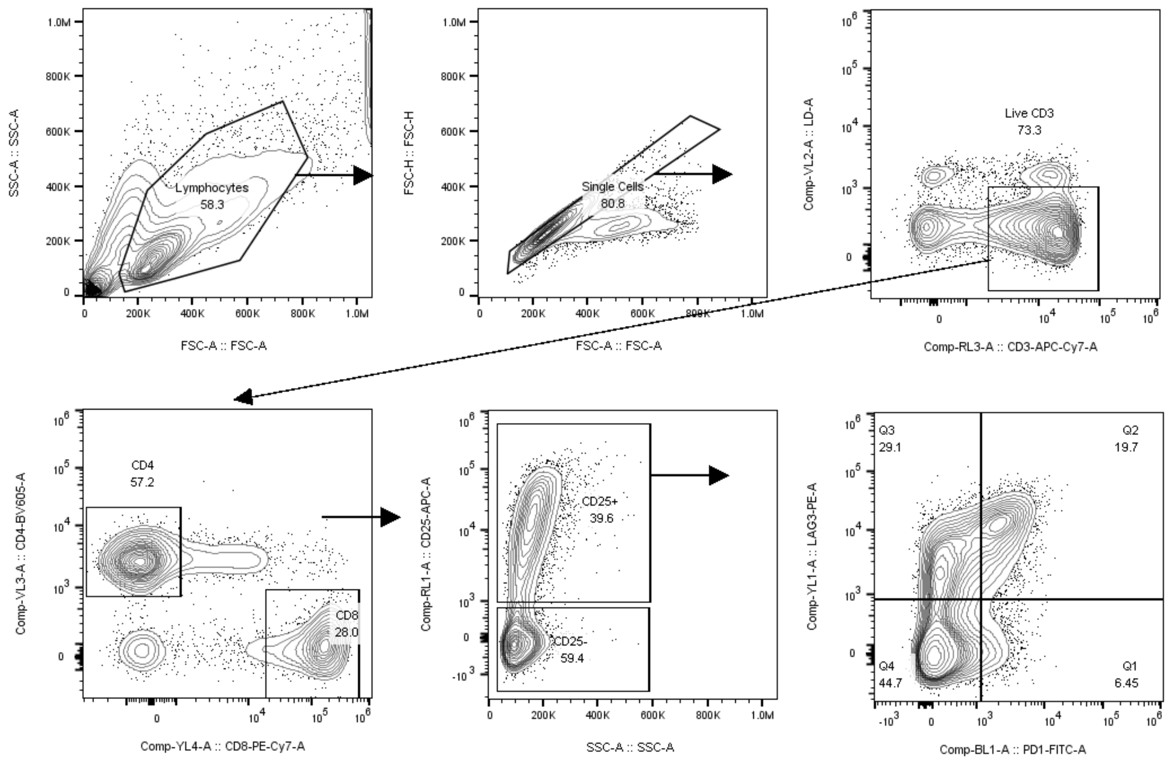

Supplementary Figure 6. Flow cytometry gating strategy. Cells were first gated for lymphocytes using SSC-A and FSC-A, then single cells were selected using FSC-H and FSC-A, then live CD3+ cells were selected using Live/Dead vs CD3-APC-CY7, CD4+ or CD8+ cells were then selected either by CD4-BV605 vs CD8-PE-Cy7, then either CD25+ or CD25- cells were selected by using CD25-APC vs SSC-A, finally cells were gated using LAG3-PE vs PD1-FITC.

Supplementary Information:

Methods for single-cell RNA sequencing and analyses

200,000 PBMCs were cultured in 200µL cRPMI in the presence or absence of 1µL ImmunoCult Human CD3/CD28 T Cell activator (*STEMCELL Technologies,* #10971) for 24 hours. Eight samples from different individuals were pooled and loaded onto two separate lanes on a Chromium X (10x genomics) at 18,250 cells per individual sample per lane (at a total number of 146,000 cells per lane) using Chromium Next GEM Single Cell 3' HT Kit v3.1 (10x Genomics, #1000348) and Chromium Next GEM Chip G Single Cell Kit (10x Genomics (10x Genomics, #1000120) following manufacturer's instructions.

Post GEM-RT Cleanup and cDNA Amplification

All procedures were according to manufacturer's instructions (Chromium Next GEM Single Cell 3' HT Reagent Kits v3.1, Dual Index).

In short, a Recovery agent was added to the GEMs, resulting in a biphasic mixture. Following the removal of the pink oil partition, the clear aqueous phase was incubated with the Dynabead Cleanup mix, which contained Cleanup Buffer, Dynabeads MyOne SILANE (Thermo Fisher) and Reducing Agent B. The mixure was vortexed and incubated for 10 min at room temperature, followed by magnetic bead separation using the 10X Magnetic Separator HT magnet as described in the procedure. The final eluate (35 µL) was mixed with 65 µL of cDNA Amplification mix containing cDNA primers and incubated in an MJR thermal cycler using 12 cycles of amplification (72 °C) for 1 min, 15 sec (98 °C) of denaturation and 20 sec (63 °C) of annealing, respectively. Amplified cDNA was purified using 0.6X SPRIselect beads (Beckman Coulter) and eluted in 40 µL of EB buffer. Each biological sample was prepared in two separate tubes and the final cDNA sample used for downstream library preparation was made by mixing 15 uL from each cDNA tube. Quality assessment and quantitation of each cDNA library was performed using the LabChip GX with the DNA 5K reagent kit/chip (Perkin Elmer). Final cDNA samples were stored at -20 °C until further use.

3' Gene Expression Library Preparation

Fragmentation, End Repair & A-tailing. cDNA (20 µL) was added to 15 µL EB buffer and 15 µL of Fragmentation mix and stored on ice. The mixture was placed in a thermal cycler and incubated for 5 min at 32 °C (fragmentation), followed by 30 min at 65 °C (end-repair and A-

trailing). The mixture was purified and size selected using magnetic SPRIselect beads at 0.6X and 0.8X bead:sample ratios, respectively. Final elution was done in EB buffer (50 µL).

Adaptor Ligation. Adaptor ligation mix was prepared by mixing ligation buffer, DNA ligase and adaptor oligos. Samples and ligation mix were mixed in equal volumes (50 µL:50 µL) and incubated on the thermal cycles for 15 min at 20 °C. Samples were purifed using SPRIselect at 0.8X ratio and eluted in 30 µL of EB buffer.

Sample Index PCR. Indexes/barcodes were added to each sample using the dual index Plate TT Set A. Samples (30 µL) were added to 50 µL AMP Mix and the appropriate dual index (20 µL). Samples were incubated in the thermal cycler using 12 cycles of amplification (72 °C) for 1 min, 20 sec (98 °C) of denaturation and 30 sec (54 °C) of annealing, respectively. Amplified and indexed samples were size selected using the double-sided SPRIselect method as described above (0.6X and 0.8X bead:sample ratios). Final purified sequencing libraries were eluted in 35 µL of EB buffer and stored at -20 °C. Libraries were quantified using the LabChip GX as described above.

Sequencing

Samples were pooled appropriately in equimolar amounts and sequenced on the Illumina NovaSeq6000 sequencer using the XP workflow (individual lane loading) for the S4 flowcell (4-lanes). Samples were sequenced using paired-end dual index sequencing with the following number of cycles:

Read1: 28 cycles (10X barcode and UMI)

Read2: 10 cycles (i7 index read)

Read3: 10 cycles (i5 index read)

Read4: 90 cycles (3‘ sample read)

BCL data was collected locally and copied to a central NFS storage system for further processing.All sample handling, procedures and run information were registered and stored in an in-house LIMS.

Single cell RNA sequencing analysis

Sequenced reads were aligned using CellRanger 7.0.0 and demultiplexed using genetic variants and SoupOrCell v2.0[76]. Quantification of gene expression was done using kallisto

0.48.0[58] and bustools 0.41.0[77] on the demultiplexed cells and downstream processing of the data was done in SCANPY 1.9.1[78]. Gene expression was computed within each cell type by normalizing w.r.t. read counts within the cell type.

To compute the effect of the rs781745126-T variant and stimulation we used the following mixed-effect model

log(y) ~ stim + g + stim:g + sex + age + (1|ind)

where y is the expression of LAG3, stim is the simulation condition (0, no stimulation, 1 CD3/CD28), g is the allele count of the variant and (1|ind) is a random intercept for each individual. The interaction term, stim:g captures the effect of the variant on the response to stimulation. We performed the regression of the mixed-effect model using the lme4 R package, version 1.1-33 and P-values computed using the lmerTest package version 3.1-3 and Satterthwaite's method.

Cell types were assigned based on marker genes (Supplementary Data 13) using the following process. Briefly, within each sample the mean and variance expression of each gene was computed and the associated count converted into a Pearson residual. In the first step for each cell, a cell identity was assigned based on the gene with the highest residual on the primary gene list. If a marker gene is shared between two cell types, we consider the second highest person residual on the gene list for the respective cell types. Second, for cells where no cell identity was assigned in the first step, they are assigned the cell identity using the majority vote of their nearest neighbours. In the third step, each cell is assigned the cell identity of their nearest neighbours by majority vote. Additional marker genes are extracted for cell identity, c, by selecting the top 10 genes that maximize the product of

$$P_c \cdot (1 - max_{c' \neq c} P_{c'}) \cdot \frac{O_{C-} E_C}{E_c}$$

where P_c is the proportion of cells assigned to cell identity c that express the gene and O_c is the observed fraction of reads of the gene that comes from cells assigned to c, and E_c is the expected fraction of reads that come from cells assigned to c if the reads of the gene were distributed equally. Using the additional marker genes, we repeat steps 1-3 to compute new cell identities for each cell.

After this, we compute a mean expression vector (MEV) of all genes for each cell identity and compute the distance from each cell to an MEV using the Kullback-Leibler divergence.

The MEV closest to cell determines the final assigned cell identity. Finally, for monocytes, B-cells and dendritic cells we excluded any cells that showed expression of CD2 or CD3 (Supplementary Data 13).
