# Supplementary material for: Start codon variant in LAG3 is associated with decreased LAG-3 expression and increased risk of autoimmune thyroid disease
Source: Nat Commun. 2024 Jul 9;15:5748. doi: 10.1038/s41467-024-50007-7 (PMC11233504; doi:10.1038/s41467-024-50007-7)
Supplement: Supplementary file 3 — Description of Additional Supplementary Files [file 41467_2024_50007_MOESM3_ESM.pdf]

Description of Additional Supplementary Files

File Name: Supplementary Data 1
Description: Sequence variants that are associated with autoimmune thyroid disease in genome-wide association meta-analysis on 110,945 cases and 1,084,290 controls from Iceland, Finland, UK and USA.

File Name: Supplementary Data 2
Description: Previously reported associations of AITD lead signals or correlated variants (r2>0.8) in genome-wide association studies (GWAS).

File Name: Supplementary Data 3
Description: Sequence variants associated with autoimmune thyroid disease in genome-wide association meta-analysis or correlated variants (r2>0.8) that point to candidate genes through an effect on protein coding.

File Name: Supplementary Data 4
Description: Sources of the mRNA expression (eQTL) data used in the systematic variant annotation of the lead AITD variants.

File Name: Supplementary Data 5
Description:. Association of autoimmune thyroid disease (AITD) associated signals with mRNA expression in various tissues based on findings from 18 data sources.

File Name: Supplementary Data 6
Description: Effect of autoimmune thyroid disease (AITD) associated signals on mRNA splicing (sQTL) in various tissues.

File Name: Supplementary Data 7
Description: Plasma proteins measured on the SomaScan platform that are associated with autoimmune thyroid disease (AITD) associated signals or correlated variants (r2>0.8) in a proteome-wide association study (pQTL) on Icelanders.

File Name: Supplementary Data 8
Description: Plasma proteins measured on the Olink platform that are associated with autoimmune thyroid disease (AITD) associated signals or correlated variants (r2>0.8) in a proteome-wide association study (pQTL) on 49 thousand participants in the UK Biobank.

File Name: Supplementary Data 9
Description: Results for sequence variants that are associated with AITD in GWAS meta-analysis (presented in Supplementary Data 1), shown separately for each of the four study populations, with and without conditional analysis.

File Name: Supplementary Data 10
Description: Association of sequence variants in LAG3 that are enriched in Iceland or Finland, with autoimmune phenotypes or with cancer.

File Name: Supplementary Data 11
Description: Association of LAG3 rs781745126-T with cancer subtypes and survival in Iceland.

File Name: Supplementary Data 12
Description: Effect of rs781456126-T on mRNA expression of LAG3 in peripheral blood mononuclear cell (PBMC) subpopulations in relation to stimulation (anti-CD3/anti-CD28).

File Name: Supplementary Data 13
Description: Marker genes and cell type classification of single-cell RNA (scRNA) sequencing data.
