# Supplementary material for: Start codon variant in LAG3 is associated with decreased LAG-3 expression and increased risk of autoimmune thyroid disease
Source: Nat Commun. 2024 Jul 9;15:5748. doi: 10.1038/s41467-024-50007-7 (PMC11233504; doi:10.1038/s41467-024-50007-7)
Supplement: Supplementary file 5 — Reporting Summary [file 41467_2024_50007_MOESM5_ESM.pdf]

nature portfolio

Corresponding author(s): *Saedis Saevarsdottir and Kari Stefansson*

Last updated by author(s): *Jun 3, 2024*

Reporting Summary

Nature Portfolio wishes to improve the reproducibility of the work that we publish. This form provides structure for consistency and transparency in reporting. For further information on Nature Portfolio policies, see our Editorial Policies and the Editorial Policy Checklist.

Statistics

For all statistical analyses, confirm that the following items are present in the figure legend, table legend, main text, or Methods section.

n/a	Confirmed	
☐	☒	The exact sample size (*n*) for each experimental group/condition, given as a discrete number and unit of measurement
☐	☒	A statement on whether measurements were taken from distinct samples or whether the same sample was measured repeatedly
☐	☒	The statistical test(s) used AND whether they are one- or two-sided<br>*Only common tests should be described solely by name; describe more complex techniques in the Methods section.*
☐	☒	A description of all covariates tested
☐	☒	A description of any assumptions or corrections, such as tests of normality and adjustment for multiple comparisons
☐	☒	A full description of the statistical parameters including central tendency (e.g. means) or other basic estimates (e.g. regression coefficient) AND variation (e.g. standard deviation) or associated estimates of uncertainty (e.g. confidence intervals)
☐	☒	For null hypothesis testing, the test statistic (e.g. *F*, *t*, *r*) with confidence intervals, effect sizes, degrees of freedom and *P* value noted<br>*Give P values as exact values whenever suitable.*
☒	☐	For Bayesian analysis, information on the choice of priors and Markov chain Monte Carlo settings
☒	☐	For hierarchical and complex designs, identification of the appropriate level for tests and full reporting of outcomes
☐	☒	Estimates of effect sizes (e.g. Cohen's *d*, Pearson's *r*), indicating how they were calculated

Our web collection on statistics for biologists contains articles on many of the points above.

Software and code

Policy information about availability of computer code

Data collection	*No software was used for data collection.*
Data analysis	*We used publicly available codes and software in conjunction with the methods described in the manuscript, that are available under the following URLs:<br>Graphtyper version 2, https://github.com/DecodeGenetics/graphtyper<br>PANTHER v.16.0, http://www.pantherdb.org/tools/<br>Variant Effect Predictor (release 100), https://github.com/Ensembl/ensembl-vep<br>IMPUTE2 version 2.3.1, https://mathgen.stats.ox.ac.uk/impute/impute_v2.html<br>dbSNP version 140, http://www.ncbi.nlm.nih.gov/SNP/<br>STAR software package, version 2.7.10, https://github.com/alexdobin/STAR<br>Ensembl version 87, https://www.ensembl.org/index.html<br>LeafCutter version 1 , https://github.com/davidaknowles/leafcutter<br>kallisto version 0.46, https://github.com/pachterlab/kallisto<br>Eagle https://alkesgroup.broadinstitute.org/Eagle/<br>ADMIXTURE v1.23 http://www.genetics.ucla.edu/software<br>PLINK v.190b3a http://pngu.mgh.harvard.edu/purcell/plink/<br>UMAP https://github.com/diazale/umap_review<br>GORpipe https://github.com/gorpipe/gor<br>UCSC Browser https://genome.ucsc.edu/<br>COLOC software package https://cran.r-project.org/web/packages/coloc/vignettes/a01_intro.html<br>Alphafold: https://github.com/google-deepmind/alphafold*

GWAS catalog https://www.ebi.ac.uk/gwas/
We used R, version 3.6.0 to analyze data and create plots: https://www.r-project.org/, https://ggplot2.tidyverse.org/
No custom code was written for this study.

For manuscripts utilizing custom algorithms or software that are central to the research but not yet described in published literature, software must be made available to editors and reviewers. We strongly encourage code deposition in a community repository (e.g. GitHub). See the Nature Portfolio guidelines for submitting code & software for further information.

Data

Policy information about availability of data

All manuscripts must include a data availability statement. This statement should provide the following information, where applicable:

- Accession codes, unique identifiers, or web links for publicly available datasets
- A description of any restrictions on data availability
- For clinical datasets or third party data, please ensure that the statement adheres to our policy

The data with sequence variants passing GATK filters in our previously described Icelandic population WGS data have been deposited at the European Variant Archive database under accession number PRJEB15197 (https://www.ebi.ac.uk/ena/data/view/PRJEB15197). The GWAS summary statistics are available in Supplementary Data and at https://www.decode.com/summarydata/. FinnGen data are publicly available and were downloaded from https://www.finngen.fi/en/access_results. The UK Biobank data were downloaded under application no. 56270. The meta-analysis association results and other data supporting the findings of this study are available within the article, in Supplementary Data or Source Data. Proteomics data and protein mapping to UniProt identifiers and gene names were provided by SomaLogic and Olink and the results are provided in Supplementary Data.
The authors declare that the data supporting the findings of this study are available within the article, in supplementary files or at https://www.decode.com/summarydata/.

Research involving human participants, their data, or biological material

Policy information about studies with human participants or human data. See also policy information about sex, gender (identity/presentation), and sexual orientation and race, ethnicity and racism.

Reporting on sex and gender

The association analysis is done on males and females combined, with trait values adjusted for sex differences with sex determined by genetic analysis.

Reporting on race, ethnicity, or other socially relevant groupings

In the association analysis, we used data on individuals of European descent, as determined by genetic ancestry analysis, as the trait measurements available included only few individuals not belonging to those groups. Within the Icelandic, UK Biobank and USA populations additional population substructure was adjusted for by adjusting the measurements either by genetic principle components (UK and USA) or county of origin (Iceland).

Population characteristics

Our study is based on data from study participants of European descent from four populations (Iceland, UK, USA, finnland). A description of all population characteristics is included in the methods section. Genetic ancestry filtering and principal components determining European ancestry in each population are also described in methods.
The Icelandic dataset is based on whole-genome sequence data from 63,460 Icelanders participating in various research projects at deCODE genetics. Variants identified through whole-genome sequencing were imputed into 173,025 chip-genotyped Icelanders as well as their untyped close relatives based on genealogy. This resulted in a study population of 346,753 individuals, including cases with AITD, other autoimmune diseases or with cancer, identified at Landspitali, the National University Hospital of Iceland (the only tertiary care hospital in Iceland), since 1977, and from the Registers of Primary Health Care Contacts and of Contacts with Medical Specialists in Private Practice (since 2010). This includes measurement of thyroid autoantibodies from the only department of clinical immunology in Iceland, available from 2005. Information about cancer diagnoses were retrieved from the nationwide Icelandic Cancer Registry.
The UK Biobank study is a large prospective cohort study of around 500,000 individuals, who enrolled in the study between 2006 and 2010 throughout the UK and were aged 40-69 years at recruitment. Of those, 431,079 were genotypically verified of white British (Caucasian) origin and serve as basis for the current study. Variants imputed into UK Biobank samples were derived from whole-genome sequencing of 131,958 UK individuals, performed jointly by deCODE genetics and the Wellcome Trust Sanger Institute.
In the USA, Intermountain Healthcare is a Utah-based healthcare system of 24 hospitals and 160 clinics. In a collaboration project, samples collected by Intermountain have been genotyped at deCODE genetics. A subset of 16,661 individuals were whole-genome sequenced. The imputation dataset included 79,085 samples identified to be of Caucasian origin using ancestry analysis. See Methods for more detailed description of these cohorts.
In Finland, the FinnGen research project has provided publicly available GWAS results for numerous phenotypes. The study collected samples from biobanks in Finland and phenotype data at national health registries. For information on genotyping in FinnGen, see online documentation: https://finngen.gitbook.io/documentation/methods/genotype-imputation.

Recruitment

For the Icelandic dataset individuals were recruited through various research projects at deCODE genetics. The participants are a large fraction of the adult Icelandic population and phenotypes retrieved through nationwide registries (see above and for phenotypes below). In the USA, Intermountain Healthcare is a Utah-based healthcare system of 24 hospitals and 160 clinics. Participants in UK Biobank were recruited through assessment centres, designed specifically for this purpose. In Finland, the FinnGen study collected samples from biobanks in Finland and phenotype data at national health registries.
Cases definitions: Individuals who had received a diagnosis of Graves' disease (E05.9) or Hashimoto's thyroiditis (E06.3) were considered AITD cases as well as those who had been diagnosed with other hypothyroidism (E03.9) and/or had received thyroxin-treatment (ATC-code H03AA01), excluding known non-autoimmune causes of hypothyroidism (thyroid cancer, drug-induced hypothyroidism (E03.2 or ATC-drug codes for lithium (N05AN01), amiodarone (C01BD01) and interferon (L03AB) treatments)).
Using this approach, there is unlikely a self-selection bias in the identification of cases in the cohort studies included. In the UK

Biobank study the medical history of all participants was reviewed. I
We tested the lead signals in AITD in other autoimmune diseases if the total number of cases was over 500 cases in meta-analyses of the same study populations (or 100 cases in the Icelandic or Finnish cohorts, for the population specific variants). Cases were defined by clinical diagnoses and/or ICD10 codes: type 1 diabetes (E10), celiac disease (K900), systemic lupus erythematosus (M329), rheumatoid arthritis (M058, M059, M060, M068, M069) or it's seropositive (M058, M059) and seronegative (M060, M068) subsets (defined by ICD10 codes or by positivity for rheumatoid factor and/or anti-CCP antibodies, as previously described)12; multiple sclerosis (G35), ankylosing spondylitis (M45), Sjögren's syndrome (M350), inflammatory bowel disease (K50, K51) and it's subsets ulcerative colitis (K51) and Crohn's disease (K50), psoriasis (L40), psoriatic arthritis (L405/M073) and primary biliary cirrhosis (K473), vitiligo (L12) and myasthenia gravis (G70).
Diagnoses of malignancies, including hematological and thyroid (thyroid cancer is excluded from the AITD phenotype), were retrieved from the cancer registries13 and databases in the study populations collecting information based on the ICD system and includes information on histology (systemized nomenclature of medicine, SNOMED).

Ethics oversight

All data and samples on which this study is based, were collected under licences obtained from the respective studies' local ethics and data privacy protection committees and under informed consent of participants, as described in detail in the methods section. In short:
In the Icelandic dataset, all genotyped participants signed a written informed consent. The study was approved by the National Bioethics Committee (approval no. VSN-16-042, VSN 17-171, VSN 18-115) following evaluation of the Icelandic Data Protection Authority.
The UK Biobank data was obtained under application number 56270. All participants provided written informed consent and The North West Research Ethics Committee reviewed and approved the UK Biobank protocol (ref. 06/MRE08/65).
In USA, the study has been approved by the Intermountain Healthcare Institutional Review Board, and all participants have provided written informed consent. The data is sourced from Intermountain INSPIRE Registry of individuals with heart disease and HerediGene, a general population study.
In Finland, all participants provided written informed consent, and the study has been approved by the Coordinating Ethics Committee of Helsinki and Uusimaa Hospital District.

Note that full information on the approval of the study protocol must also be provided in the manuscript.

Field-specific reporting

Please select the one below that is the best fit for your research. If you are not sure, read the appropriate sections before making your selection.

☒ Life sciences ☐ Behavioural & social sciences ☐ Ecological, evolutionary & environmental sciences

For a reference copy of the document with all sections, see nature.com/documents/nr-reporting-summary-flat.pdf

Life sciences study design

All studies must disclose on these points even when the disclosure is negative.

Sample size

Sample sizes for the GWAS, mRNA expression and proteomic analyses are reported in the article and correspond to the data that was available in our large data sources. No sample size calculation was performed for the hypothesis-free analyses on these large study cohorts, while we in the validation study comparing the LAG-3 plasma levels of age and sex-matched carriers and non-carriers, calculated the number needed based on the observed difference in the discovery screen. The sample size selected for the comparison of LAG-3 expression on activated lymphocyte subsets, with carriers and non-carriers matched for age and sex was decided based on numbers needed to provide meaningful differences based on previous studies.

Data exclusions

No available data was excluded, other than data from participants of non-European ethnicity as described for all cohorts in methods.

Replication

We performed GWAS studies in four independent populations and combined the results. Results are presented for the populations independently and combined and heterogeneity of effects between groups are assessed. We did not conduct replication since we had all study data available to us included in the GWAS meta-analysis.

Randomization

No randomizations were used, as this is a case-control study within a large cohort, but the logistic regression analyses were adjusted for year of birth, sex and origin or the first principal components (see methods).

Blinding

Not relevant for this study.

Behavioural & social sciences study design

All studies must disclose on these points even when the disclosure is negative.

Study description

Briefly describe the study type including whether data are quantitative, qualitative, or mixed-methods (e.g. qualitative cross-sectional, quantitative experimental, mixed-methods case study).

Research sample

State the research sample (e.g. Harvard university undergraduates, villagers in rural India) and provide relevant demographic information (e.g. age, sex) and indicate whether the sample is representative. Provide a rationale for the study sample chosen. For studies involving existing datasets, please describe the dataset and source.

Sampling strategy

Describe the sampling procedure (e.g. random, snowball, stratified, convenience). Describe the statistical methods that were used to

Sampling strategy	*predetermine sample size OR if no sample-size calculation was performed, describe how sample sizes were chosen and provide a rationale for why these sample sizes are sufficient. For qualitative data, please indicate whether data saturation was considered, and what criteria were used to decide that no further sampling was needed.*
Data collection	*Provide details about the data collection procedure, including the instruments or devices used to record the data (e.g. pen and paper, computer, eye tracker, video or audio equipment) whether anyone was present besides the participant(s) and the researcher, and whether the researcher was blind to experimental condition and/or the study hypothesis during data collection.*
Timing	*Indicate the start and stop dates of data collection. If there is a gap between collection periods, state the dates for each sample cohort.*
Data exclusions	*If no data were excluded from the analyses, state so OR if data were excluded, provide the exact number of exclusions and the rationale behind them, indicating whether exclusion criteria were pre-established.*
Non-participation	*State how many participants dropped out/declined participation and the reason(s) given OR provide response rate OR state that no participants dropped out/declined participation.*
Randomization	*If participants were not allocated into experimental groups, state so OR describe how participants were allocated to groups, and if allocation was not random, describe how covariates were controlled.*

Ecological, evolutionary & environmental sciences study design

All studies must disclose on these points even when the disclosure is negative.

Study description	*Not relevant.*
Research sample	*Not relevant.*
Sampling strategy	*Not relevant.*
Data collection	*Not relevant.*
Timing and spatial scale	*Not relevant.*
Data exclusions	*Not relevant.*
Reproducibility	*Not relevant.*
Randomization	*Not relevant.*
Blinding	*Not relevant.*

Did the study involve field work? ☐ Yes ☒ No

Field work, collection and transport

Field conditions	*Not relevant.*
Location	*Not relevant.*
Access & import/export	*Not relevant.*
Disturbance	*Describe any disturbance caused by the study and how it was minimized.*

Reporting for specific materials, systems and methods

We require information from authors about some types of materials, experimental systems and methods used in many studies. Here, indicate whether each material, system or method listed is relevant to your study. If you are not sure if a list item applies to your research, read the appropriate section before selecting a response.

Materials & experimental systems

n/a	Involved in the study
☐	☒ Antibodies
☒	☐ Eukaryotic cell lines
☒	☐ Palaeontology and archaeology
☒	☐ Animals and other organisms
☒	☐ Clinical data
☒	☐ Dual use research of concern
☒	☐ Plants

Methods

n/a	Involved in the study
☒	☐ ChIP-seq
☐	☒ Flow cytometry
☒	☐ MRI-based neuroimaging

Antibodies

Antibodies used

FACS staining: The following antibodies were purchased from Biolegend: LAG-3 PE (#369306), PD-1 FITC (#329904), CD20 APC-Cy7 (#302314, Lymphoblasts), CD3 APC-Cy7(#300318, PBMC), CD4 BV605 (#300556, PBMC), CD8 PE-Cy7 (#301012, PBMC) and CD25 APC (#302510, PBMC).
Soluble LAG-3 in plasma and cell medium was measured by using MSD R-PLEX Human LAG3 (# F213Y-3) according to manufacturer's protocol (Meso Scale Diagnostics).

Validation

Antibodies were validated by manufacturer.

Eukaryotic cell lines

Policy information about cell lines and Sex and Gender in Research

Cell line source(s)

Not relevant.

Authentication

Not relevant.

Mycoplasma contamination

Not relevant.

Commonly misidentified lines
(See ICLAC register)

Not relevant.

Palaeontology and Archaeology

Specimen provenance

Not relevant.

Specimen deposition

Not relevant.

Dating methods

Not relevant.

☐ Tick this box to confirm that the raw and calibrated dates are available in the paper or in Supplementary Information.

Ethics oversight

Not relevant.

Note that full information on the approval of the study protocol must also be provided in the manuscript.

Animals and other research organisms

Policy information about studies involving animals; ARRIVE guidelines recommended for reporting animal research, and Sex and Gender in Research

Laboratory animals

Not relevant.

Wild animals

Not relevant.

Reporting on sex

Not relevant.

Field-collected samples

Not relevant.

Ethics oversight

Not relevant.

Note that full information on the approval of the study protocol must also be provided in the manuscript.

Clinical data

Policy information about clinical studies
All manuscripts should comply with the ICMJE guidelines for publication of clinical research and a completed CONSORT checklist must be included with all submissions.

Clinical trial registration	*Not relevant.*
Study protocol	*Not relevant.*
Data collection	*Not relevant.*
Outcomes	*Not relevant.*

Dual use research of concern

Policy information about dual use research of concern

Hazards

Could the accidental, deliberate or reckless misuse of agents or technologies generated in the work, or the application of information presented in the manuscript, pose a threat to:

No	Yes	
☒	☐	Public health
☒	☐	National security
☒	☐	Crops and/or livestock
☒	☐	Ecosystems
☒	☐	Any other significant area

Experiments of concern

Does the work involve any of these experiments of concern:

No	Yes	
☒	☐	Demonstrate how to render a vaccine ineffective
☒	☐	Confer resistance to therapeutically useful antibiotics or antiviral agents
☒	☐	Enhance the virulence of a pathogen or render a nonpathogen virulent
☒	☐	Increase transmissibility of a pathogen
☒	☐	Alter the host range of a pathogen
☒	☐	Enable evasion of diagnostic/detection modalities
☒	☐	Enable the weaponization of a biological agent or toxin
☒	☐	Any other potentially harmful combination of experiments and agents

Plants

Seed stocks	*Not relevant.*
Novel plant genotypes	*Not relevant.*
Authentication	*Not relevant.*

ChIP-seq

Data deposition

☐ Confirm that both raw and final processed data have been deposited in a public database such as GEO.

☐ Confirm that you have deposited or provided access to graph files (e.g. BED files) for the called peaks.

Data access links<br>*May remain private before publication.*	*Not relevant.*
Files in database submission	*Not relevant.*
Genome browser session<br>(e.g. UCSC)	*Not relevant.*

Methodology

Replicates	*Not relevant.*
Sequencing depth	*Not relevant.*
Antibodies	*Not relevant.*
Peak calling parameters	*Not relevant.*
Data quality	*Not relevant.*
Software	*Not relevant.*

Flow Cytometry

Plots

Confirm that:

☒ The axis labels state the marker and fluorochrome used (e.g. CD4-FITC).

☒ The axis scales are clearly visible. Include numbers along axes only for bottom left plot of group (a 'group' is an analysis of identical markers).

☒ All plots are contour plots with outliers or pseudocolor plots.

☒ A numerical value for number of cells or percentage (with statistics) is provided.

Methodology

Sample preparation	*PBMCs were isolated from venous blood samples via standard Ficoll-Paque (GE Health, #17144002) density gradient centrifugation at 800G for 15 min in 50ml Blood-Sep spin tubes (DACOS, #037100SI) and cryopreserved in liquid nitrogen. Prior to use cells where thawn and incubated over night at 37°C and 5% CO2 at 1.5x107 cells/mL in RPMI 1640 supplemented with 10% fetal bovine serum (FBS) and 1x Penicillin-Streptomycin (Gibco, #15140148) (cRPMI). After resting overnight cells were filtered, counted and seeded in a 96-well plate at 1x10^6 cells/well.*<br>*Cells were stained in U bottom 96 well plates. Cells were washed in PBS and Fc receptors blocked with TruStain FcX (Biolegend, #422302) according to manufacturer's protocol. Live/Dead fixable aqua dead cell stain (Invitrogen, #L34957) was added to the Fc block and incubated at RT for 20 min. Cells were washed with FACS buffer (PBS + 2% FBS) and stained for 20 min at RT.*
Instrument	*Attune NxT*
Software	*FlowJo*
Cell population abundance	*Describe the abundance of the relevant cell populations within post-sort fractions, providing details on the purity of the samples and how it was determined.*
Gating strategy	*Cells were first gated for lymphocytes using SSC-A and FSC-A, then single cells were selected using FSC-H and FSC-A, then live CD3+ cells were selected using Live/Dead vs CD3-APC-CY7, CD4+ or CD8+ cells were then selected either by CD4-BV605 vs CD8-PE-Cy7, then either CD25+ or CD25- cells were selected by using CD25-APC vs SSC-A, finally cells were gated using LAG3-PE vs PD1-FITC.*

☒ Tick this box to confirm that a figure exemplifying the gating strategy is provided in the Supplementary Information.

Magnetic resonance imaging

Experimental design

Design type	*Not relevant.*
Design specifications	*Not relevant.*
Behavioral performance measures	*Not relevant.*

Acquisition

Imaging type(s)	*Not relevant.*
Field strength	*Not relevant.*
Sequence & imaging parameters	*Not relevant.*
Area of acquisition	*Not relevant.*

Diffusion MRI ☐ Used ☐ Not used

Preprocessing

Preprocessing software	*Not relevant.*
Normalization	*Not relevant.*
Normalization template	*Not relevant.*
Noise and artifact removal	*Not relevant.*
Volume censoring	*Not relevant.*

Statistical modeling & inference

Model type and settings	*Not relevant.*
Effect(s) tested	*Not relevant.*

Specify type of analysis: ☐ Whole brain ☐ ROI-based ☐ Both

Statistic type for inference<br>(See Eklund et al. 2016)	*Specify voxel-wise or cluster-wise and report all relevant parameters for cluster-wise methods.*
Correction	*Not relevant.*

Models & analysis

n/a	Involved in the study
☒	☐ Functional and/or effective connectivity
☒	☐ Graph analysis
☒	☐ Multivariate modeling or predictive analysis

Functional and/or effective connectivity	*Not relevant.*
Graph analysis	*Not relevant.*
Multivariate modeling and predictive analysis	*Not relevant.*
